# Assessment of Periodontal Biotype in a Young Chinese Population using Different Measurement Methods

**DOI:** 10.1038/s41598-018-29542-z

**Published:** 2018-07-25

**Authors:** Yunmin Shao, Lanlan Yin, Jianyu Gu, Dongmiao Wang, Wei Lu, Ying Sun

**Affiliations:** 1 Jiangsu Key Laboratory of Oral Diseases, Nanjing Medical University, Nanjing, China; 2 Department of Periodontology, Affiliated Hospital of Stomatology, Nanjing Medical University, Nanjing, China; 3 Department of Oral and Maxillofacial Surgery, Affiliated Hospital of Stomatology, Nanjing Medical University, Nanjing, China

## Abstract

Periodontal biotype is used to describe the morphological characteristics of periodontal tissues and is closely related to periodontal health and prognosis of many dental treatments. This study was undertaken to explore the periodontal biotype distribution in a young Chinese population and to evaluate the accuracy of different methods for gingival thickness (GT) measurement. A total of 372 teeth from 31 periodontally healthy subjects were included. GT was measured simultaneously by probe transparency, transgingival probing and cone-beam computed tomography (CBCT). Some other anatomic parameters, including crown width/crown length ratio, attached gingival width, labial bone thickness and papilla volume were recorded for periodontal biotype classification. As found by probe transparency, the gingivae of 222 teeth (59.68%) were thick, while those of 150 teeth (40.32%) were thin. The mean GT of included subjects was 1.03 ± 0.31 mm as measured by transgingival probing and 1.03 ± 0.24 mm as measured by CBCT. Four groups were identified by cluster analysis. Thick-flap biotype, average-scalloped biotype, average-flap biotype and thin-scalloped biotype comprised 137 teeth (36.83%), 96 teeth (25.81%), 39 teeth (10.48%) and 100 teeth (26.88%), respectively. These results demonstrate that the most common periodontal biotype in this young Chinese population was the thick-flap type with low aesthetic risk.

## Introduction

Clinical appearance of gingiva differs from subject to subject and even among different teeth. It is closely related to periodontal health and the prognosis of many dental treatments. To describe the variation in gingival contour, “gingival biotype” was first proposed by Ochsenbein and Ross^1^. Then, Seibert and Lindhe introduced the term “periodontal biotype”, which classified the gingival contour into two categories, the “thick” and “thin” biotype, based on simple visual appearance of gingivae^2^. In 1997, Muller included some new parameters, such as tooth shape and gingival width, in his analysis of periodontal biotype^3^. De Rouck developed a new method for the classification of gingival biotype based on the following four clinical parameters: gingival thickness (GT), crown width/crown length ratio (CW/CL), gingival width and papilla height (PH). Then, three biotypes were identified: the thin-scalloped, thick-scalloped and thick-flat biotype^4^. Unfortunately, until now, there has been no unified criterion for periodontal biotype classification, although GT is undoubtedly the most important indicator of all for evaluation.

Many invasive and non-invasive methods have been employed to assess periodontal biotype. However, the most accurate method for periodontal biotype assessment and GT measurement has not yet been confirmed. Simple visual inspection was the first method developed to evaluate periodontal biotype, but it was not considered to be a reliable method by many researchers^5,6^. Transgingival probing is a traditional invasive method with limited application in clinic. Instead, another evaluation that is based on the transparency of the periodontal probe through the gingival margin is widely used and is taken as a simple method with excellent repeatability. Ultrasonic measurement and cone beam computerized tomography (CBCT) are also non-invasive methods, but special devices are needed for these assessments. Moreover, it was reported that the accuracy of GT measurement by ultrasonic instruments was worse than that by direct puncture^7^. Comparisons of different measurement methods based on the same sample are limited.

Some anatomic parameters, such as CW/CL, attached gingival width (AGW), PH, bone thickness (BT) and tooth site, were reported to be related to GT^4,8,9^. Different results of GT assessment and periodontal biotype exploration in different studies were also due to the included samples with different age, sex and racial characteristics^3,4,8,10–12^. Until recently, most research on periodontal biotype has been about Caucasians. In contrast, explorations in Chinese populations are limited.

The aim of the present study was to explore the periodontal biotype distribution in a young Chinese population and to evaluate the accuracy of different methods for GT measurement, including probe transparency, transgingival probing and CBCT.

## Method and Materials

### Subjects

This study was performed in compliance with the revised Helsinki Declaration and was approved by the Ethical Committee of Nanjing Medical University (Permit Number: 20160206). Written informed consent was obtained from all recruits.

A total of 31 periodontally healthy students in the College of Stomatology, Nanjing Medical University (aged between 18 and 27 years, mean age 22.2 years, 15 males and 16 females) were enrolled in this study from October 2016 to February 2017. Inclusion criteria were: (1) 18–30 years old; (2) no site with gingival index (GI) ≥1, probing depth (PD) >3 mm or clinical attachment loss (CAL) ≥1 mm and no radiographic evidence of alveolar bone loss; and (3) no malocclusion, crowding, missing or supernumerary anterior teeth. Exclusion criteria were: (1) crown restorations or fillings in anterior teeth; (2) previous orthodontic treatment; (3) previous periodontal surgery, including gingivectomy, flap surgery, guided tissue regeneration or periodontal plastic surgery; (4) systemic disease; (5) pregnancy and lactation; (6) use of any drugs that might lead to gingival enlargement during the past 6 months; (7) smoking; and (8) bruxism. All volunteers received oral hygiene instructions and a full-mouth supragingival scaling 1 week before clinical examination to control any possible gingival inflammation.

### Clinical measurement

A total of 372 anterior teeth from 31 subjects, including upper and inferior anterior teeth, were involved in this study. Clinical periodontal parameters, including plaque index (PLI), GI, PD and CAL were recorded at six sites (mesial labial, midlabial, distal labial, mesial palatal, midpalatal and distal palatal) per tooth. All measurements were performed by a calibrated examiner.

For transgingival probing, local anesthesia (4% articaine) was applied over the gingiva of anterior teeth. To avoid mucous swelling due to local anesthesia, clinical measurements were taken 20 min after anesthesia. GT by transgingival probing (GTp) was determined at the level of the cementoenamel junction (CEJ) by piercing midfacial gingival with a #15 endodontic K-file (MANI, Tochigi, Japan), which had a rubber stop. Gingivae thicker than 0.8 mm were defined as thick, while others were categorized as thin^13^.

The following anatomic parameters were measured by a Williams probe (Hu-Friedy, IL, USA) to the nearest 0.5 mm:

1. CW/CL. Crown length was recorded as the distance between the incisal edge of the tooth and the free gingival margin, or if discernible, the CEJ. Crown width was the distance between the approximal tooth surfaces, which was measured at the border between the middle and the cervical third of the crown.
2. AGW was evaluated at the midfacial point by subtracting PD from keratinized gingival width, which was the distance from the free gingival margin to the mucogingival junction.
3. PH was defined as the distance from the tip of the papilla to a line connecting the midfacial soft tissue margin of two adjacent teeth.

Moreover, the visibility of a probe inserted through the gingival margin at the midfacial site of the tooth was also determined to assess periodontal biotype. If the outline of the probe could be seen through the gingiva, it was defined as thin. Otherwise, it was regarded as thick.

### Radiographic measurement

To avoid the interference of surrounding soft tissues (lip and tongue), acrylic plates of maxillary and mandibular arches extending beyond the mucogingival junction were made, and zinc oxide–eugenol cement was loaded into it. Then, the plates were allowed to set intraorally during the process of CBCT examination.

**Figure 1:**
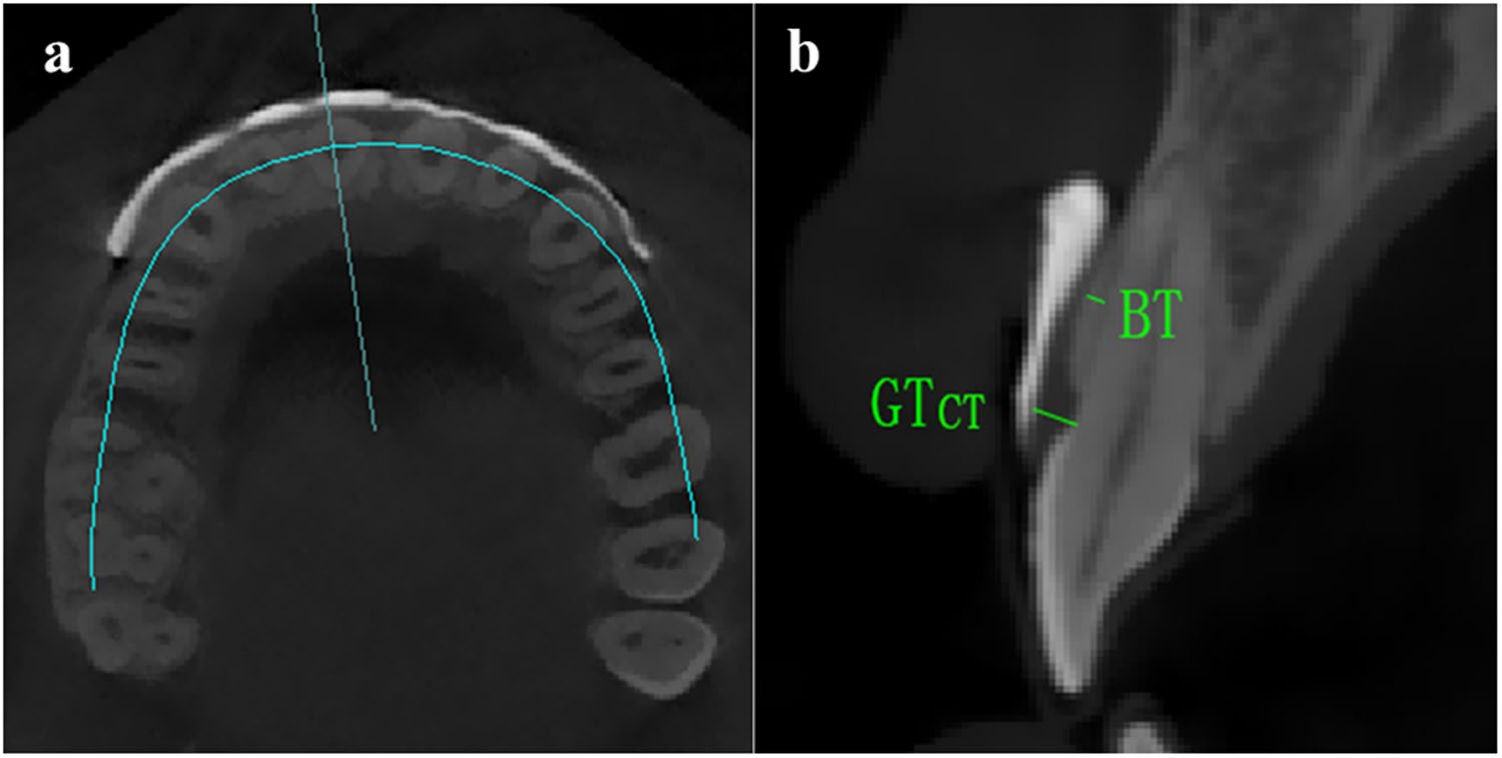
Measurements of GT_CT_ and BT in a cross-sectional image. GT_CT_ and BT were measured perpendicular to the long axis of the tooth (**a**). GT_CT_ at CEJ and BT at the midpoint of the root were measured at the midfacial surface in a cross-sectional image that passed through the long axis of the tooth (**b**).

**Figure 2:**
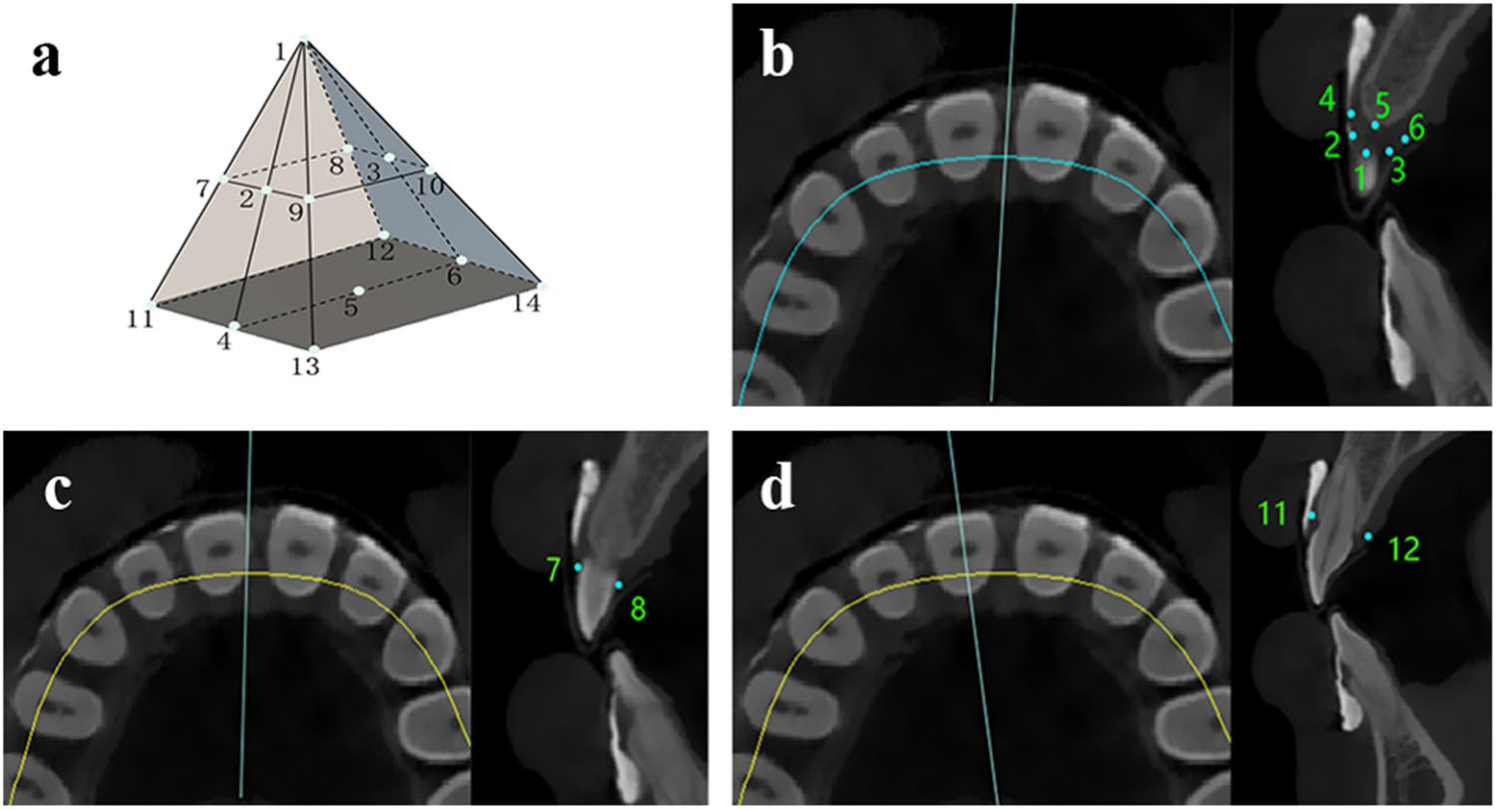
3D imaging reconstruction and the measurements of PV. After CBCT scanning and 3D imaging reconstruction, interdental papilla was defined as a quadrangular pyramid outlined by 14 points (**a**). Points 1–6 were located in the mid-cross-sectional plane perpendicular to the connecting line of two neighbouring teeth (**b**). Points 7–10 were located at the free gingival margin in the proximal surfaces of the teeth (**c**). Points 11–14 were located in the cross-sectional image that passed through the long axis of the tooth and was perpendicular to labial surfaces (**d**).

CBCT scanning was performed using NewTom 5G (version FP, Verona, Italy). Exposure was performed at 110 kV in 4–5 mA for 3.6 s. Reconstructed images were generated, and digital measurements were taken using Mimics software (Materialise NV, Leuven, Belgium) with an accuracy of 0.01 mm. The following parameters were recorded: (1) GT in CBCT scanning (GT_CT_) was measured at the level of CEJ; (2) labial bone thickness (BT) was assessed at the midpoint of the root. The above-mentioned thickness measurements were conducted perpendicular to the midfacial surface of the tooth, as shown in Fig. 1; and (3) after 3D imaging reconstruction, gingival papilla volume (PV), which was defined by 14 points, was calculated automatically by Mimics software (Fig. 2).

### Intra-examiner repeatability

All measurements were performed by one examiner (YMS). 10 volunteers randomly extracted from the total samples were reevaluated after1 month by the same clinician.

### Statistical analysis

Statistical analysis of clinical and radiologic parameters, including GT, CW/CL, AGW, PH and BT, was performed using Student’s t-test. Spearman’s correlation coefficient was used to analyze the consistency of GT obtained by transgingival probing and CBCT, as well as the correlations of CW/CL, AGW, PH, BT, PV and GT. Agreement of probe transparency and GT measurements was analyzed by kappa test. Hierarchical cluster analysis (HCA) based on Euclidian distances of four parameters, including GT_P_, CW/CL, AGW and PV, was conducted to develop periodontal tissues typology more objectively. Before the parameters were put into HCA, they were standardized in order to ensure the same weight in the analysis. Differences in morphometric variables of the resultant clusters were analyzed by ANOVA, and the LSD test was used to compare differences between groups.

### Data availability

The data generated and analyzed in this present study are available from the corresponding author upon reasonable request.

## Results

### Intra-examiner accuracy control

The accuracy and repeatability of the measurements were repeatedly evaluated in 10 volunteers. Pearson correlation coefficients were 0.92 (p < 0.001), 0.84 (p < 0.001), 0.94 (p < 0.001), 0.85 (p < 0.001), 0.76 (p < 0.001) and 0.72 (p < 0.001) for CW/CL, AGW, PH, GT_P_, GT_CT_ and BT, respectively. Probe transparency also proved to be highly reproducible, with 86% agreement between duplicate measurements (k = 0.80, p < 0.05).

### Clinical and radiographic parameters

Descriptive characteristics of 4 clinical parameters (CW/CL, AGW, PH and GT_P_) and 3 radiographic parameters (GT_CT_, BT and PV) are presented in Table 1. The mean CW/CL, AGW, PH, GT_P_, GT_CT_, BT and PV were 0.77, 5.37 mm, 4.34 mm, 1.03 mm, 1.03 mm, 0.67 mm and 75.21 mm^3^, respectively. Significant differences between males and females were found in CW/CL, GT_P_ and BT (p < 0.05) and differences between maxillary and mandibular teeth were found in all of the 7 variables (p < 0.05) (Table 2).

**Table 1:** Descriptive characteristics of clinical and radiographic parameters (mean ± SD).

	Male	Female	Maxillary	Mandibular	Total	Min–Max
CW/CL	0.75 ± 0.13	0.78 ± 0.13*	0.84 ± 0.10	0.69 ± 0.10^†^	0.77 ± 0.13	0.50–1.22
AGW (mm)	5.41 ± 1.52	5.34 ± 1.33	5.95 ± 1.41	4.79 ± 1.19^†^	5.37 ± 1.42	2.50–10.00
PH (mm)	4.47 ± 0.87	4.22 ± 0.78	4.60 ± 0.89	4.08 ± 0.69^†^	4.34 ± 0.84	2.00–7.00
GT_P_ (mm)	1.06 ± 0.32	0.99 ± 0.30*	1.21 ± 0.27	0.85 ± 0.24^†^	1.03 ± 0.31	0.30–1.82
GT_CT_ (mm)	1.04 ± 0.26	1.02 ± 0.23	1.21 ± 0.25	0.94 ± 0.21^†^	1.03 ± 0.24	0.55–1.68
BT (mm)	0.64 ± 0.23	0.69 ± 0.20*	0.73 ± 0.22	0.60 ± 0.17^†^	0.67 ± 0.21	0.00–1.92
PV (mm^3^)	77.98 ± 25.57	72.61 ± 24.79	90.58 ± 21.71	59.84 ± 18.30^†^	75.21 ± 25.27	19.83–149.33

CW/CL crown width/crown length ratio, AGW attached gingival width, PH papilla height, GT_p_ gingival thickness measured by transgingival probing, GT_CT_ gingival thickness measured by CBCT, BT bone thickness, and PV papilla volume.

*p < 0.05 compared with males, ^†^p < 0.05 compared with maxillary teeth.

**Table 2:** Effects of tooth position on clinical and radiographic parameters (mean ± SD).

	11/21	12/22	13/23	31/41	32/42	33/43
CW/CL	0.82 ± 0.07	0.81 ± 0.09	0.90 ± 0.10	0.62 ± 0.07*	0.68 ± 0.07*	0.76 ± 0.08*
AGW (mm)	6.00 ± 1.32	6.04 ± 1.36	5.81 ± 1.45	5.01 ± 1.25*	4.95 ± 1.12*	4.42 ± 0.95*
PH (mm)	5.16 ± 0.89	4.44 ± 0.74	4.48 ± 0.77	3.94 ± 0.70*	3.86 ± 0.55*	4.38 ± 0.52
GT_P_ (mm)	1.36 ± 0.24	1.16 ± 0.19	1.10 ± 0.23	0.89 ± 0.23*	0.84 ± 0.24*	0.83 ± 0.20*
GT_CT_ (mm)	1.26 ± 0.19	1.02 ± 0.17	1.08 ± 0.18	0.94 ± 0.17*	0.94 ± 0.16	0.95 ± 0.17*
BT (mm)	0.75 ± 0.15	0.71 ± 0.19	0.74 ± 0.22	0.64 ± 0.15*	0.56 ± 0.15*	0.61 ± 0.12*
PV (mm^3^)	105.51 ± 21.49	80.36 ± 15.51	93.33 ± 19.20	46.47 ± 9.38*	51.45 ± 9.58*	74.91 ± 10.29*

CW/CL crown width/crown length ratio, AGW attached gingival width, PH papilla height, GT_p_ gingival thickness measured by transgingival probing, GT_CT_ gingival thickness measured by CBCT, BT bone thickness, and PV papilla volume.

*p < 0.05 compared with homonym tooth in maxillary.

### Comparisons of GT measured by different methods

In this present research, GT was measured by transgingival probing, probe transparency and CBCT in each subject at the same time. Distribution of thick/thin gingiva as assessed by different methods is shown in Table 3. The Spearman’s correlation coefficient of GT_P_ and GT_CT_ was 0.40 (p < 0.001), which revealed a moderate correlation between these two methods. The kappa value of probe transparency and transgingival probing was 0.24 (p < 0.001), which indicated an unsatisfactory consistency. Moreover, no consistency was discovered between probe transparency and CBCT (k = 0.09, p = 0.051).

**Table 3:** Distribution of thick/thin gingiva assessed by different methods.

	Thin gingiva	Thick gingiva
Probe transparency	40.32% (150/372)	59.68% (222/372)
Transgingival probing	28.49% (106/372)	71.51% (266/372)
CBCT	18.55% (69/372)	81.45.2% (303/372)

GTp or GT_CT_ ≥ 0.8 mm was defined as thick gingiva, while the remainder were categorized as thin.

### Correlation analysis of clinical and radiographic parameters

Correlation analysis was carried out to explore the factors closely related to periodontal biotype. A strong correlation between CW/CL and PV was revealed (p < 0.01, r = 0.554), while moderate correlations between CW/CL-AGW, CW/CL-GT_P_, AGW-GT_P_, AGW-GT_CT_, CT_CT_-GT_P_, PV-GT_P_, GT_CT_-PV and PV-PH were also found (p < 0.01, 0.3 < r ≤ 0.5). In addition, weak correlations existed between CW/CL-PH, CW/CL-GT_CT_, CW/CL-BT, AGW-BT, AGW-PV, PH-GT_P_, PH-BT, GT_P_-BT, GT_CT_-BT and BT-PV (p < 0.01, 0 < r ≤ 0.3). There was no significant correlation between AGW-PH and PH-GT_CT_ (p > 0.05) (Table 4).

**Table 4:** Correlation analysis of clinical and radiographic parameters (Spearman’s rho).

	CWCL	AGW	PH	GT_P_	GT_CT_	BT	PV
CWCL	1	0.328*	0.161*	0.406*	0.286*	0.183*	0.554*
AGW	0.328*	1	0.029	0.384*	0.308*	0.150*	0.285*
PH	0.161*	0.029	1	0.182*	0.083	0.196*	0.435*
GT_P_	0.406*	0.384*	0.182*	1	0.428*	0.258*	0.416*
GT_CT_	0.286*	0.308*	0.083	0.428*	1	0.264*	0.342*
BT	0.183*	0.150*	0.196*	0.258*	0.264*	1	0.226*
PV	0.554*	0.285*	0.435*	0.416*	0.342*	0.226*	1

CW/CL crown width/crown length ratio, AGW attached gingival width, PH papilla height, GT_p_ gingival thickness measured by transgingival probing, GT_CT_ gingival thickness measured by CBCT, BT bone thickness, and PV papilla volume.

*p < 0.001.

### Cluster analysis

**Figure 3:**
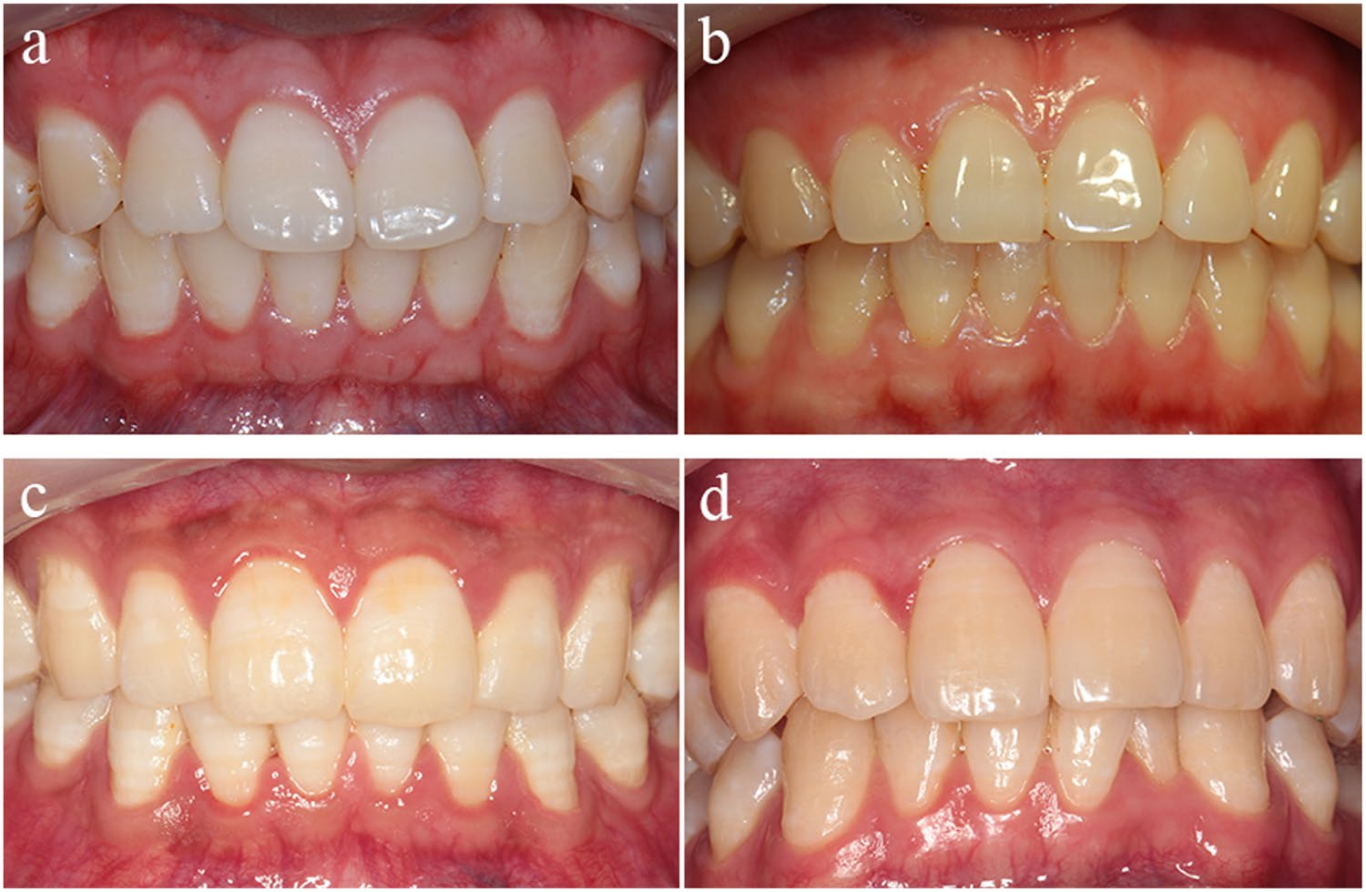
Representative photographs of the four periodontal biotypes. 11 in (**a**) 11 in (**b**) 11 in (**c**) and 22 in (**d**) were thick-flap, average-scalloped, average-flap and thin-scalloped biotype respectively.

By HCA, four clusters of periodontal biotype were identified based on GT_P_, CW/CL, AGW and BT. Cluster A (thick-flap type), cluster B1 (average-scalloped type), cluster B2 (average-flap type) and cluster C (thin-scalloped type) included 137 teeth (36.83%), 96 teeth (25.81%), 39 teeth (10.48%) and 100 teeth (26.88%), respectively. Representative photographs of these four periodontal biotypes are presented in Fig. 3.

Among the four groups, the GT_P_ of cluster A was the thickest (p < 0.001), while that of cluster C was the thinnest (p < 0.001). No significant differences in GT_P_ were found between cluster B2 and B1 (p > 0.05). The CW/CL of cluster B2 was significantly larger than those of the other groups (p < 0.001), which represented a quarter tooth shape. Similarly, the PVs of clusters A and B2 were much greater than those of clusters B1 and C (p < 0.001). Furthermore, the AGWs of clusters B1 and C were significantly smaller than those of the other two groups (p < 0.001) (Table 5).

**Table 5:** Clinical parameters per cluster (mean ± SD).

	Cluster A	Cluster B1	Cluster B2	Cluster C	Total
n	137	96	39	100	372
CW/CL	0.84 ± 0.10*^,†,‡^	0.72 ± 0.07^†,‡^	0.92 ± 0.10‡	0.65 ± 0.09	0.77 ± 0.13
AGW (mm)	6.00 ± 1.17*^†,‡^	4.31 ± 0.84^†^	7.21 ± 1.20^‡^	4.82 ± 1.10	5.37 ± 1.42
PH (mm)	4.56 ± 0.89^†,‡^	4.39 ± 0.75^‡^	4.67 ± 1.09	4.00 ± 0.72	4.34 ± 0.84
GT_P_ (mm)	1.27 ± 0.20*^,†,‡^	1.02 ± 0.26^‡^	1.01 ± 0.26^‡^	0.71 ± 0.17	1.03 ± 0.31
CT_CT_ (mm)	1.13 ± 0.26^*,‡^	0.99 ± 0.22	1.07 ± 0.26	0.93 ± 0.19	1.03 ± 0.24
BT (mm)	0.72 ± 0.25	0.65 ± 0.20	0.65 ± 0.19^‡^	0.63 ± 0.15	0.67 ± 0.21
PV (mm^3^)	89.98 ± 26.22*^,‡^	69.20 ± 20.24^†,‡^	90.24 ± 23.65^‡^	55.36 ± 18.18	75.21 ± 25.27

CW/CL crown width/crown length ratio, AGW attached gingival width, PH papilla height, GT_p_ gingival thickness measured by transgingival probing, GT_CT_ gingival thickness measured by CBCT, BT bone thickness, and PV papilla volume.

*p < 0.001 in comparison with clusterB1, ^†^p < 0.001 in comparison withclusterB2, ^‡^p < 0.001 in comparison with cluster C.

## Discussion

Periodontal biotype is closely related to the prognosis of many dental treatments, especially in the anterior aesthetic area. After implantation, periodontal therapy or orthodontic treatment, severe gingival recession may develop more easily in patients with a thin biotype, while deep periodontal pockets are more likely to form in persons with a thick biotype^14^. Therefore, it is important to identify periodontal biotype accurately in order to predict prognosis and avoid unexpected complications.

In this present study, the GT in a young Chinese population was evaluated by different methods (probe transparency, transgingival probing and CBCT) and the periodontal biotype distribution was explored by cluster analysis. Moreover, PV was first measured by CBCT and its relationship with periodontal biotype was analyzed at the same time.

The influences of some factors, such as the position and shape of the tooth, race, gender and age, on periodontal biotype have been confirmed by some researchers^3,4^. Until now, most studies on periodontal biotype have been in Caucasians. In Stein’s study, 60 central maxillary incisors from 60 periodontally healthy Caucasian were involved, and the mean GT was 1.25 mm^11^. In Nikiforidou’s research, 42 periodontally healthy subjects were recruited, and the mean GT of 186 maxillary anterior teeth was found to be 1.20 mm^12^. Le’s study included 84 maxillary teeth from 14 periodontally healthy Chinese individuals, and revealed that subjects with thin biotypes were only 9% of that population^15^. In addition, in most studies, only maxillary anterior teeth were involved. Differences in GT between maxillary and mandibular anterior teeth were reported by La Rocca and Muller^16,17^. Although the aesthetic appearance of mandibular anterior teeth does exist, studies concerning mandibular teeth are still limited. Therefore, both maxillary and mandible teeth were included in this study and the sample size was expanded to 372 teeth.

In the past ten years, periodontal biotype distribution was explored in numerous studies. Among them, the periodontal biotypes of subjects, but not their teeth, were confirmed by most researchers^4,10,11,18–20^. However, the effects of tooth sites on GT were revealed by Müller and Vandana^17,21^. Therefore, differences in periodontal biotype/GT from tooth to tooth in the same subject do exist. It is imprecise to classify a mandibular tooth with thin gingiva as a thick biotype based on the data from a maxillary tooth with thick gingiva. De Rouck noticed that the results of transgingival probing in two maxillary incisors might be different^4^. In this present study, differences in GT_P_, AGW, PH and BT between maxillary and mandibular teeth were also observed, which further confirmed that the periodontal biotype of upper anterior teeth might not represent the biotype of all anterior teeth, let alone the whole dentition. To avoid unsatisfactory prognosis, it is still very important to explore the biotype of inferior teeth accurately before some dental treatments, such as orthodontic treatment and implantation^22,23^. Therefore, we tried to assess periodontal biotype tooth by tooth in this study.

Many invasive and non-invasive methods have been employed to assess periodontal biotype, such as transgingival probing, probe transparency and CBCT^4,12,24,25^. Transgingival probing is an invasive direct measurement performed by inserting an injection needle, periodontal probe or endodontic file through gingiva under local anesthesia. Infusion of the anesthetic agent, angulations of probing and distortion of tissues might affect the precision of this measurement. In addition, excessive puncture strength might lead to the penetration of periosteum and even lamina dura. Probe transparency is a non-invasive method based on the transparency of a periodontal probe through the gingival margin. A clinical trial involving 100 periodontally healthy subjects indicated that probe transparency was highly reproducible, with 85% agreement between duplicate recordings^4^. However, it is also a subjective method, and its accuracy depends on the examiner’s experience. Stein found that the prognostic value of probe transparency for GT and BT was limited^11^. CBCT, a three-dimensional radiographic technique, is a routine examination for hard tissues. In a CBCT scan, similar radiographic densities of gingivae, lips, cheeks and tongue prevents the identification of gingivae, and a contrast agent, plastic lip retractor or cotton rolls are then needed to make soft tissues visible^26,27^. Moreover, gingivae are too thin to be measured accurately, and a high-resolution screen and easily operated measurement software are therefore necessary.

So far, there has been no comparison of the consistency of these three methods. In this study, an excellent consistency of transgingival probing and CBCT was confirmed, while similar consistency between transgingival probing/CBCT and probe transparency was not found in the same samples. Untill now, there has been no accurate, reliable and simple measurement method widely accepted by researchers all over the world. Dentists should choose the appropriate method according to their experiences and actual cases. For example, CBCT measurements might be included in orthodontic treatment and implantation. For these patients, it might be an excellent choice for periodontal biotype assessment.

During the past ten years, the correlation between periodontal anatomic parameters has always been focus of attention, while no consensus has been reached. Multi-parameter correlations were observed in this study, including CW/CL-GT_P_, PV-GT_P_ and AGW-GT_P_, which implied that GT was highly correlated with the tooth and its surrounding tissues. Stein^11^ and Nikiforidou^12^ also found a close relationship between CW/CL and GT at CEJ, which was similar to our results, while Fischer did not^8^. For convenience of measurement, crown length was recorded as the distance between incisal edge of the teeth and free gingival margin in this study. However, it is important to point out that, to some degree, clinical crown length (from incisal edge to free gingival margin) is different from anatomical crown length (from incisal edge to CEJ), which might affect the results. Moreover, Nikiforidou indicated that GT at the CEJ was related to labial BT at a point 3 mm apical to the CEJ^12^. However, La Rocca pointed out that BT was related to AGW, but not to GT^16^. As far as BT was concerned, different results might be due to different samples and measurement sites. Nikiforidou confirmed a moderate association between GT and BT, while it was Mallikarjun’s conclusion that there was no significant correlation between them^12,28^. Our study indicated that the correlations between BT at the midpoint of the root and some other parameters (GT_P_, CW/CL, AGW and PV) were weak. In addition, there were no differences in BT among the four groups created by cluster analysis, which suggested that BT might be less associated with periodontal biotypes.

Gingival papilla in the anterior aesthetic area is not only an important biological barrier to protect the teeth and surrounding tissues but also an artificial factor in aesthetics^29^. During treatment in the aesthetic area, attention needs to be paid not only to the health of gingivae but also to their beauty and harmony. The appearance of interdental papilla, especially its height, is important for aesthetics in implantation^30^. De Rouck reported that the mean PH in Caucasians was 3.96 mm^4^. Cao found that the mean height and width of papilla were 3.67 mm and 4.35 mm in Chinese, which was different from our results^31^. Compared with the height and width of papilla, PV is a parameter in three-dimensional space and may represent the quantity of soft tissue more accurately. It is almost impossible to measure PV without CBCT scanning and three-dimensional reconstruction. Therefore, until now, there have been no studies exploring PV. Our study first found that PV was highly correlated with CW/CL and GT_P_, which implied the underlying relationship between PV and the appearance of tooth and periodontal soft tissues. In this present study, smaller PV was found to be more common in teeth characterized by average-scalloped and thin-scalloped biotypes. Black triangles caused by the destruction of papilla are a major cosmetic deficiency for patients who expect aesthetically pleasing restorative or orthodontic treatments. Some researchers found that black triangles developed more easily in patients with scalloped teeth^32^. It is unclear that whether PV is a risk factor for black triangles, and the relationship between PV and black triangles needs to be further explored.

Exploration of periodontal biotype distribution by cluster analysis was undertaken by some researchers. K-mean clustering algorithm, which classified samples according to categories specified by investigators, was employed by Müller, De Rouck and Gobbato, who artificially classified the included subjects into three periodontal biotypes^4,24,25^. Nikiforidou found four periodontal types by HCA^12^. During this statistical analysis, numbers of categories were decided by the characteristics of the data themselves, not artificially imposed by researchers. When a small sample is analyzed, the results by HCA may be scattered and difficult to generalize, while k-mean clustering may be efficient and easy to derive results. However, when larger samples are analyzed, HCA can reflect the characteristics of the data themselves as much as possible. Therefore, owing to the quantity of teeth included in this study, HCA was employed.

In papers that employed cluster analysis, different variables were analyzed in different studies, and their weights were also different. In general, GT and the shape of teeth have been the most important and frequently employed parameters to distinguish periodontal biotypes^11^. Gobbato even paid so much attention to tooth morphology that he did not consider GT and BT as parameters for cluster analysis^24^. Some other variables, including AGW, keratinized gingiva width and PH, were also taken into consideration by some studies^4,25^. Due to the strong correlations of PV-CW/CL and PV-GT_P_, we included PV in cluster analysis for the first time.

As a result of the variety of included samples, measurement means and statistical methods, different categories and proportional distribution were obtained in different papers. In Stein’s study, 46.7% of Caucasians were classified as having the thin biotype, and the others were classified as having the thick biotype^11^. De Rouck included 100 Caucasians and thin-scalloped, thick-flat and thick-scalloped biotypes were found to be 37%, 29% and 34% of the sample, respectively^4^. Le’s study identified that the thin, compromised and thick biotypes in Chinese individuals were 9%, 50% and 41%, respectively^15^. Four periodontal biotypes were reported by Nikiforidou: thin, average, mixed and thick type, which comprised 31.9%, 31.3%, 16.6% and 20.2% of the sample, respectively^12^. In our results, the most and the least common types were thick-flat (36.8%) and average-flat type (10.5%), while the rest were average-scalloped and thin-scalloped type (each approximately 1/4 of the total samples). Our present study also revealed an effectively equal distribution of slender and square teeth in a young Chinese population (slender 52.69% and square 47.31%). Moreover; there were more slender teeth in subjects with average/thin gingival and more square teeth in persons with thick/average gingiva.

In summary, a possible diversity of periodontal biotypes beyond thick and thin might exist in the young Chinese population. The most common periodontal biotype was the thick-flat biotype, which displayed a square tooth, average AGW, thick BT and large gingival papilla. Meanwhile, the second-largest biotype, the thin-scalloped biotype, which displayed a slender tooth, narrow AGW, thin BT and small gingival papilla, was the one with the highest aesthetic risk. In clinical practice, more attention should be paid to these teeth, especially in the aesthetic zone.
